# Admission blood glucose as a predictor of shock and mortality in multiply injured patients

**DOI:** 10.1051/sicotj/2019015

**Published:** 2019-05-28

**Authors:** Marcel Winkelmann, Ada Luise Butz, Jan-Dierk Clausen, Richard David Blossey, Christian Zeckey, Sanjay Weber-Spickschen, Philipp Mommsen

**Affiliations:** 1 Trauma Department, Hannover Medical School Carl-Neuberg-Strasse 1 30625 Hannover Germany; 2 Department of General, Trauma and Reconstructive Surgery, Ludwig-Maximilians-University Munich Marchioninistrasse 15 81377 Munich Germany

**Keywords:** Shock, Blood glucose, Multiple trauma, Hemorrhage

## Abstract

*Introduction*: Reliable diagnosis of shock in multiply injured patients is still challenging in emergency care. Point-of-care tests could have the potential to improve shock diagnosis. Therefore, this study aimed to analyze the impact of admission blood glucose on predicting shock in multiply injured patients.

*Methods*: A retrospective cohort analysis of patients with an injury severity score (ISS) ≥ 16 who were treated in a level I trauma center from 01/2005 to 12/2014 was performed. Shock was defined by systolic blood pressure ≤ 90 mmHg and/or shock index ≥ 0.9 at admission. Laboratory shock parameters including glucose were measured simultaneously. Receiver-operating-characteristic (ROC) analysis and multivariate logistic regression analysis was performed.

*Results*: Seven hundred and seventy-two patients were analyzed of whom 93 patients (12.0%) died. Two hundred and fifty-nine patients (33.5%) were in shock at admission. Mortality was increased if shock was present at admission (18.1% vs. 9.0%, *p* < 0.001). Mean glucose was 9.6 ± 4.0 mmol/L if shock was present compared to 8.0 ± 3.0 mmol/L (*p* < 0.001). Admission glucose positively correlated with shock (Spearman rho = 0.2, *p* < 0.001). Glucose showed an AUC of 0.62 (95% CI [0.58–0.66], *p* < 0.001) with an optimal cut off value of 11.5 mmol/L. Patients with admission glucose of > 11.5 mmol/L had a 2.2-fold risk of shock (95% CI [1.4–3.4], *p* = 0.001). Admission blood glucose of > 11.5 mmol/L positively correlated with mortality too (Spearman rho = 0.65, *p* < 0.001). Patients had a 2.5-fold risk of dying (95% CI [1.3–4.8], *p* = 0.004).

*Discussion*: Admission blood glucose was proven as an independent indicator of shock and mortality and, therefore, might help to identify multiply injured patients at particular risk.

## Introduction

Multiply injured patients are at particular risk for hemorrhagic shock. Shock, in turn, is a major cause of death in trauma patients [1]. Therefore, early detection of shock in multiply injured patients is of particular importance. In a recent study, Mutschler et al. demonstrated the adequacy of the shock index, a quotient of heart frequency and systolic blood pressure, in predicting shock in multiply injured patients [2]. The calculation is easy and the measures have to be collected anyway. However, there are some shortcomings in practice, especially in prehospital care. Blood pressure measurement exceedingly depends on examiners [3, 4]. External circumstances at the accident scene can additionally hamper a reliable measurement especially in multiply injured patients. Thus, assessment of hypovolemic shock at scene is unreliable [5]. A simple point-of-care testing, however, could play its strengths in prehospital and emergency care, whenever standard circulatory monitoring is problematic or unreliable. Blood glucose measurement is a well-established practice. Its wide availability and ease of use are substantial advantages. Blood sugar imbalances are common in critically ill patients and hyperglycemia could be demonstrated as an indicator of poor outcome and mortality in trauma patients [6, 7]. In two recent studies Kreutziger et al. reported blood glucose to be a predictor of hemorrhagic shock in trauma patients [8, 9]. However, definitions of shock and injury severity are inconsistent which in turn limits generalizability.

Therefore, this study aimed to analyze the impact of admission blood glucose on predicting shock in a precisely defined cohort of multiply injured patients.

## Materials and methods

### Study design

Following institutional review board approval (No. 3392-2016), a retrospective cohort analysis was performed.

### Study setting and population

Multiply injured patients [injury severity score (ISS) ≥ 16] primarily admitted to our level I trauma center between January 2005 and December 2014 were included. Patients had to have a minimum age of 15 years to include the majority of adolescent patients. All patients underwent precisely defined trauma management at the emergency department in line with the ATLS principles, the S3 guideline “Polytrauma” and the 2nd revised and updated edition of the “Whitebook Medical Care of the Severely Injured” [10, 11].

### Measurements

Heart frequency was determined by means of electrocardiogram. Systolic blood pressure was measured by either oscillometric method or, if applicable, invasive arterial measurement. Systolic blood pressure (SBP; in mmHg) and heart rate (HR; in min^−1^) were used to calculate the shock index (SI = HR/SBP). A shock index between 0.5 and 0.7 is deemed to be physiologic [2]. Laboratory shock parameters including pH value, base excess (BE; in mmol/L), lactate (in mmol/L), bicarbonate (HCO_3_; mmol/L), glucose (in mmol/L), and hemoglobin (Hb; in g/dL) were determined by means of blood gas analysis (ABL800 FLEX blood gas analyzer, Radiometer GmbH, Krefeld, Germany). Coagulation parameters including international normalized ratio (INR), prothrombin time (PTT; in s), and thrombocytes (in ×10^9^/L) were measured with standard laboratory tests. Coagulopathy was diagnosed if at least one of the following conditions was met: INR > 1.4 or PTT > 37 s. Demographic and baseline data including age, sex, mortality, duration of mechanical ventilation, duration of intensive care as well as overall in-patient care, and transfusion requirements [packed red blood cells (PRBC), fresh frozen plasma (FFP) and platelet concentrate (PC)] were collected from the electronic patient records. Injury pattern and organ related severity were classified by a single consultant specially trained in trauma surgery using the 2008 update of the abbreviated injury scale (AIS) [12]. Overall injury severity was calculated using the injury severity score (ISS) [13]. Shock was defined by at least one of the following parameters: systolic blood pressure ≤ 90 mmHg or shock index ≥ 0.9.

### Data analysis

Statistical analyses were performed with IBM SPSS (Version 24, IBM, Armonk, NY, USA) [14]. For the comparison of plasma concentrations and other continuous variables (duration of intensive care unit stay, etc.) an analysis of variance (ANOVA) was performed. Variables with a Gaussian distribution were analyzed using parametric tests (Student’s *t*-test) and other variables were analyzed using non-parametric tests (Mann–Whitney test for independent data). Fisher’s exact test (exact *χ* ^2^ test) was used in the analysis of contingency tables. Diagnostic values of shock indicators were estimated by means of receiver-operating-characteristic (ROC) analysis and the area under the curve (AUC). Cut-off points were defined on the basis of the Youden index (*J*) and the optimal cut-off point (c*) that optimized the differentiation ability when equal weight is given to sensitivity and specificity [15]. Validity of glucose as shock indicator was additionally analyzed with threshold values according to Kreutziger et al. (≤9.4 to >9.4 mmol/L) as well as two-hour oral glucose tolerance standard value in compliance with the latest diabetes guideline (<7.8 to ≥7.8 mmol/L) [8, 16]. Multivariate logistic regression analysis on predicting shock with optimal cut off values on the basis of the Youden index (*J*) of glucose, base excess, pH, hemoglobin, HCO_3_, and lactate as well as age (per year), sex (male/female), and ISS (per point) was performed and odds ratios (OR) and 95-percent confidence intervals (95% CI) were calculated. The significance level was set at *p* < 0.05.

## Results

**Figure 1:**
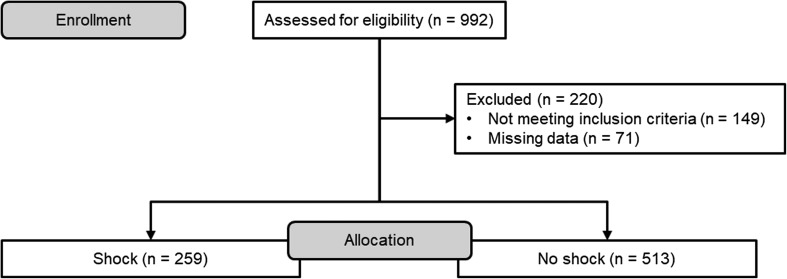
Flow diagram of excluded patients.

After checking for eligibility criteria as well as missing data 772 patients remained for further analyses (see also Figure 1). Patients had a mean age of 44.2 ± 19.4 years and a mean ISS of 29.4 ± 10.2. Five hundred and fifty-four patients (71.8%) were male. Mean time from accident to arrival was 86 ± 83 min. Ninety-three patients (12.0%) died within course of in-hospital treatment. Eight patients died due to hemorrhagic shock alone. Thirty-eight patients died because of traumatic brain injury alone. Eleven patients died due to a combination of hemorrhagic shock and traumatic brain injury. Thirty-one patients died in the course of a multiple organ dysfunction syndrome. Five patients died on any other grounds. Death due to hemorrhagic shock – also in combination with traumatic brain injury – predominantly occurred within the first 48 h (18/19 patients). Two hundred and fifty-nine patients (33.5%) were in shock at admission. There were no differences regarding age (42.4 ± 18.6 vs. 45.1 ± 19.7 years, *p* = 0.08) and sex [male sex 180 (69.5%) vs. 374 (72.9%), *p* = 0.4]. Patients with shock were injured more severely (ISS 32.3 ± 11.6 vs. 27.9 ± 9.2, *p* < 0.001). Two hundred and six patients (26.7%) presented with coagulopathy at admission. Coagulopathy was more present in patients with shock [104 (40.2%) vs. 102 (19.9%), *p* < 0.001]. Hence, early and total transfusion requirement was increased (*p* < 0.001). Patients with shock had to be ventilated longer (342 ± 340 vs. 221 ± 300 h, *p* < 0.001). Thus, intensive care (17.8 ± 15.0 vs. 12.9 ± 13.7 days, *p* < 0.001) as well as in-patient care stay (28.0 ± 23.9 vs. 22.3 ± 17.6, *p* = 0.005) was prolonged. Forty-seven patients (18.1%) with shock at admission died during in-patient care compared to 46 patients (9.0%, *p* < 0.001) without shock. Table 1 gives an overview of demographic and baseline data.

**Table 1:** Demographic, baseline and outcome data of multiply injured patients with and without shock at admission.

	Total [*n* = 772]	Shock+ [*n* = 259]	Shock− [*n* = 513]	*p*
Age [years], mean ± *SD*	44.2 ± 19.4	42.4 ± 18.6	45.1 ± 19.7	0.08^a^
Male sex, *n* (%)	554(71.8)	180(69.5)	374(72.9)	0.4^b^
Injure Severity Score (ISS)				
Median(IQR)	27(14)	29(19)	27(13)	<0.001^a^
Mean ± *SD*	29.4 ± 10.2	32.3 ± 11.6	27.9 ± 9.2	
Abbreviated Injury Scale (AIS)				
AIS_Head_, mean ± *SD*	2.2 ± 1.7	2.2 ± 1.7	2.2 ± 1.7	0.6^a^
AIS_Face_, mean ± *SD*	1.0 ± 1.2	0.9 ± 1.2	1.0 ± 1.2	0.2^a^
AIS_Thorax_, mean ± *SD*	2.8 ± 1.5	3.1 ± 1.4	2.7 ± 1.5	0.002^a^
AIS_Abdomen_, mean ± *SD*	1.2 ± 1.5	1.4 ± 1.5	1.1 ± 1.4	0.01^a^
AIS_Extremities_, mean ± *SD*	2.2 ± 1.3	2.5 ± 1.4	2.0 ± 1.3	<0.001^a^
AIS_External_, mean ± *SD*	0.9 ± 1.0	1.0 ± 1.1	0.8 ± 0.9	0.003^a^
Duration of intensive care [days], mean ± *SD*	14.5 ± 14.3	17.8 ± 15.0	12.9 ± 13.7	<0.001^a^
Duration of in-patient care [days], mean ± *SD*	24.2 ± 20.1	28.0 ± 23.9	22.3 ± 17.6	0.005^a^
Duration of mechanical ventilation [hours], mean ± *SD*	261 ± 319	342 ± 340	221 ± 300	<0.001^a^
Transfusion requirement				
PRBC 48 h [units], mean ± *SD*	6.9 ± 10.8	11.2 ± 13.8	4.8 ± 8.1	<0.001^a^
FFP 48 h [units], mean ± *SD*	5.4 ± 8.6	8.7 ± 10.5	3.7 ± 6.9	<0.001^a^
PC 48 h [units], mean ± *SD*	0.9 ± 1.9	1.4 ± 2.4	0.6 ± 1.6	<0.001^a^
PRBC total [units], mean ± *SD*	12.2 ± 16.8	18.3 ± 19.5	9.2 ± 14.4	<0.001^a^
FFP total [units], mean ± *SD*	7.7 ± 12.7	11.9 ± 15.5	5.7 ± 10.5	<0.001^a^
PC total [units], mean ± *SD*	1.2 ± 3.3	1.8 ± 4.2	0.8 ± 2.6	<0.001^a^
Mortality, *n* (%)	93(12.0)	47(18.1)	46(9.0)	<0.001^b^
Glucose [mmol/L], mean ± *SD*	8.7 ± 3.5	9.6 ± 4.0	8.0 ± 3.0	<0.001^a^
Base excess [mmol/L], mean ± *SD*	−2.5 ± 5.0	−3.8 ± 6.3	−1.8 ± 4.0	<0.001^a^
pH, mean ± *SD*	7.32 ± 0.12	7.29 ± 0.15	7.34 ± 0.09	<0.001^a^
Hemoglobin [g/dL], mean ± *SD*	11.6 ± 2.5	10.8 ± 2.6	12.1 ± 2.3	<0.001^a^
Lactate [mmol/L], mean ± *SD*	3.0 ± 2.4	3.7 ± 2.8	2.7 ± 2.1	<0.001^a^
HCO_3_ [mmol/L], mean ± *SD*	22.7 ± 4.0	21.5 ± 4.5	23.3 ± 3.6	<0.001^a^
Systolic blood pressure [mmHg], mean ± *SD*	119 ± 30	92 ± 20	133 ± 25	<0.001^a^
Heart frequency [min^−1^], mean ± *SD*	92 ± 21	105 ± 23	85 ± 16	<0.001^a^
Shock index, mean ± *SD*	0.84 ± 0.41	1.20 ± 0.52	0.66 ± 0.14	<0.001^a^
Thrombocytes [×10^9^/L], mean ± *SD*	196 ± 72	196 ± 77	196 ± 69	0.6^a^
INR, mean ± *SD*	1.3 ± 0.7	1.5 ± 0.9	1.3 ± 0.5	<0.001^a^
PTT [s], mean ± *SD*	35.3 ± 23.1	40.6 ± 28.4	32.6 ± 19.4	<0.001^a^
Coagulopathy, *n* (%)	206(26.7)	104(40.2)	102(19.9)	<0.001^b^

a Mann–Whitney *U*-test.

b Fisher’s exact test.

SD = standard deviation, PRBC = packed red blood cells, FFP = fresh frozen plasma, PC = platelet concentrate, 48 h = within 48 h following admission, INR = international normalized ratio, PTT = partial thromboplastin time.

**Figure 2:**
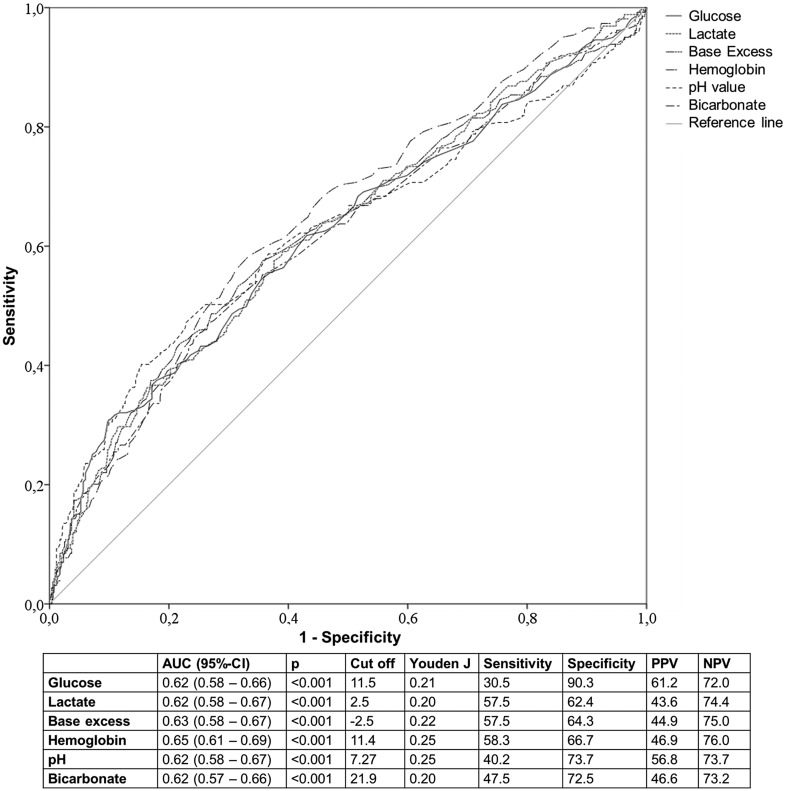
Receiver operating characteristic (ROC) curve and area under the curve (AUC) with 95% confidence interval (95% CI) of clinical and laboratory indicators of shock at admission in multiply injured patients; cut off value by means of Youden index (*J*) with sensitivity, specificity, positive predictive value (PPV), and negative predictive value (NPV); SBP = systolic blood pressure.

Patients with shock at admission showed increased values of all laboratory shock parameters (*p* < 0.001) (see also Table 1). Mean glucose was 9.6 ± 4.0 mmol/L if shock was present compared to 8.0 ± 3.0 mmol/L (*p* < 0.001). Admission glucose positively correlated with shock (Spearman rho = 0.2, *p* < 0.001). In the ROC curve analysis glucose showed an AUC of 0.62 (95% CI [0.58–0.66], *p* < 0.001). The calculated optimal cut off value was 11.5 mmol/L with a Youden *J* of 0.21. Prediction of shock was possible with a sensitivity of 30.5% and a specificity of 90.3%. The positive predictive value was 61.0% and the negative predictive value was 72.0%. Thus, glucose had the lowest sensitivity but highest specificity of all laboratory shock parameters. For further details on ROC curve and cross tab analyses of laboratory shock parameters, please refer to Figure 2. A multivariate regression analysis on predicting shock was performed. Patients with admission glucose of more than 11.5 mmol/L had a 2.2-fold risk of shock (95% CI [1.4–3.4], *p* = 0.001). Apart from glucose, hemoglobin (OR = 2.0, 95% CI [1.4–2.8], *p* < 0.001), pH (OR = 1.7, 95% CI [1.1–2.7], *p* = 0.02), age (per year: OR = 0.99, 95% CI [0.98–1.0], *p* = 0.046), and ISS (per point: OR = 1.02, 95% CI [1.0–1.04], *p* = 0.02) could be identified as indicators of shock at admission (see also Table 2). When using 9.4 mmol/L as the threshold value of admission glucose according to Kreutziger et al. sensitivity was 40.2% and specificity was 78.6%. In a multivariate regression analysis we obtained an odds ratio of 1.6 (95% CI [1.1–2.3], *p* = 0.02). When using 7.8 mmol/L as the threshold value sensitivity increased to 61.8% while specificity decreased to 56.9%. Odds ratio was 1.5 (95% CI [1.0–2.1], *p* = 0.03). For a better overview, please refer to Table 3.

**Table 2:** Multivariate regression analysis with odds ratio (OR) and 95% confidence interval (95% CI) of clinical and laboratory indicators of shock at admission in multiply injured patients.

	OR (95% CI)	*p*
Age [per year]	0.99 (0.98–1.0)	0.046
Sex [male/female]	1.06 (0.7–1.5)	0.8
ISS [per point]	1.02 (1.0–1.04)	0.02
Glucose [≤11.5 to >11.5 mmol/L]	2.2 (1.4–3.4)	0.001
Base excess [≥2.5 to ≤2.5 mmol/L]	1.3 (0.8–2.0)	0.4
pH [>7.27 to ≤7.27]	1.7 (1.1–2.7)	0.02
Hemoglobin [>11.4 to ≤11.4 g/dL]	2.0 (1.4–2.8)	<0.001
HCO_3_ [>21.4 to ≤21.4 mmol/L]	1.2 (0.7–1.9)	0.5
Lactate [≤2.5 to >2.5 mmol/L]	1.4 (1.0–2.0)	0.06

**Table 3:** Different cut off values of admission blood glucose with corresponding odds ratio (OR) and 95% confidence interval (95% CI) for predicting shock at admission in multiply injured patients with sensitivity and specificity.

	Shock+	Shock−	Sensitivity	Specificity	OR (95% CI)	*p*
Glucose ≤ 9.4 mmol/L [*n* = 630]	155	403	40.2	78.6	1.6 (1.1–2.3)	0.02
Glucose > 9.4 mmol/L [*n* = 226]	104	110				
Glucose ≤ 11.5 mmol/L [*n* = 722]	180	463	30.5	90.3	2.2 (1.4–3.4)	0.001
Glucose > 11.5 mmol/L [*n* = 134]	79	50				
Glucose < 7.8 mmol/L [*n* = 453]	99	292	61.8	56.9	1.5 (1.0–2.1)	0.03
Glucose ≥ 7.8 mmol/L [*n* = 403]	160	221				

Admission blood glucose of > 11.5 mmol/L positively correlated with mortality (Spearman rho = 0.65, *p* < 0.001). Patients with that elevated glucose level at admission had a 2.5-fold risk of dying (95% CI [1.3–4.8], *p* = 0.004) with a sensitivity of 45.2% and specificity of 87.2%. Apart from glucose age (per year: OR = 1.05, 95% CI [1.03–1.06], *p* < 0.001), ISS (per point: OR = 1.11, 95% CI [1.08–1.15], *p* < 0.001), and coagulopathy (OR = 3.6, 95% CI [1.9–6.7], *p* < 0.001) independently predicted mortality in a multivariate regression analysis. None of the additional laboratory shock parameters reached significance.

## Discussion

This study aimed to analyze the impact of admission blood glucose on predicting shock in multiply injured patients. In conclusion we could demonstrate admission glucose (cut off value = 11.5 mmol/L) to be an independent indicator of shock (OR = 2.2) and mortality (OR = 2.5). Sensitivity was 30.5% and specificity was 90.3%, respectively.

This study’s patient population is similar to that of other hospitals with the same level of care, since level I trauma centers usually provide the necessary medical care of most severely injured patients in a particular region. Following this, mortality is comparable when all multiply injured patients independently from specific injury patterns are factored into evaluation. Causes of death are predominantly traumatic brain injury, multiple organ dysfunction syndrome and hemorrhagic shock. This is in accordance with the current literature [8, 17]. In accordance with Kreutziger et al. admission blood glucose proved to be an independent indicator of mortality in multiply injured patients. Odds ratio was lower compared to the data reported by Kreutziger et al. (admission blood glucose 10–15 mmol/L: OR = 9.7, 95% CI [2.2–42.4], *p* < 0.001). This might be in part due to a higher mortality (19.5%), which in turn is only partially reflected by a mean ISS of 30.1 ± 11.1 [18]. In comparison, mean ISS of the presented patient population was 29.4 ± 10.2 and mortality totaled 12.0%.

Just like Kreutziger et al. in their 2015 analysis we could verify admission blood glucose as an independent indicator of shock [8]. However, correlation of elevated blood glucose and shock was only weak and there are significant differences in threshold value of glucose, odds ratios as well as sensitivity and specificity. The weak correlation implies a multifactorial genesis of shock. Blood glucose is only partially capable of indicating shock. Causality is possible; however, impact is limited. This reduces the clinical usefulness of the test. Additionally, we have no reliable information about the potential change of blood glucose levels during preclinical care and depending on preclinical procedures and resuscitation. The influence of the specific point in time of the blood glucose measurement on the ability of blood glucose in predicting shock is as yet unknown. Thus, we were not able validate the diagnostic performance of Kreutziger et al. in our study population, who reported an odds ratio of 10.2 (95% CI [5.4–19.2]) and a sensitivity of 66.1% and specificity of 83.9% with a cut off value of ≥ 9.4 mmol/L. Using the same cut off value we calculated an odds ratio of 1.6 (95% CI [1.1–2.3]). Sensitivity (40.2%) and specificity (78.6%) were also lower (see also Table 3). In contrast, our threshold or cut-off value according to the Youden index was even higher (>11.5 mmol/L). Nevertheless, neither odds ratio (2.2, 95% CI [1.4–3.4]) nor sensitivity (30.5%) reached that level reported by Kreutziger et al. [8]. Only specificity (90.3%) was higher. The positive (61.0%) as well as negative predictive values (72.0%) were only moderate. The test was neither able to identify nor to rule out patients with shock at admission with sufficiently high assurance. Although comparable basic statement regarding the predictive value of admission blood glucose on shock there are substantial differences in capability compared to Kreutziger’s results. There are several influencing factors that might explain these differences. While Kreutziger et al. excluded patients with known pre-injury diagnosis of diabetes mellitus or immunologically compromising diseases or therapies, we made a conscious decision to include these patients. There are two main reasons for this. First, we are convinced that a simple point-of-care test should be applicable for all multiply injured patients. The exclusion of a relevant proportion of trauma patients would impair the practical benefit of such a test. Second of all, usually there is only little information about pre-existing conditions and medication of trauma patients during pre-hospital and emergency care. Thus, the influence of diabetes is simply incalculable. Even, Kreutziger et al. did not report how many patients had to be excluded due to diabetes or immunologically compromising diseases and therapies. Another reason for the differences in capability might be heterogeneous definitions of shock. This can lead to varying numbers of patients in shock and certainly influences the prognostic value of shock parameters. The assessment of hypovolemic shock at scene by means of PHTLS classification could be demonstrated unreliable [5]. In contrast, shock index seems to reflect hypovolemic shock more closely [2]. Therefore, we decided to use a simple definition of shock (systolic blood pressure ≤ 90 mmHg or shock index ≥ 0.9) that allows a simultaneous measurement and a better attribution. Kreutziger et al., in contrast, defined shock by at least two of the following criteria within the first 12 h following admission: systolic blood pressure < 90 mmHg, use of catecholamines to increase perfusion pressure, blood loss > 20% of estimated body blood volume, requirement of a mass transfusion (substitution of at least the amount of the patient’s estimated blood volume with blood products), or lactate acidosis (≥2 mmol/L) [8]. In our opinion, there are substantial confounders. Catecholamine use is not clearly defined. Thus, vasoactive drug use is mainly due to an individual decision of the attending physician. The potentially highest impact, however, can be attributed to the long period of time (12 h) in which mass transfusion as well as catecholamine use can occur. The necessity of mass transfusion as well as vasoactive drug use can be due to iatrogenic blood loss during surgery either. Usually a lot of therapeutic decisions will be made within 12 h following admission of a multiply injured patient. Each decision has the potential to influence the patient’s hemodynamic situation. Therefore, it seems questionable to associate a fluctuating laboratory parameter measured at admission with a hemodynamic impairment that occurred several hours later. In order to rule out this risk, we decided to measure outcome parameter and potential indicators of outcome at one time. Nevertheless, hemodynamics is developing dynamically. Analyzing shock at admission is therefore a necessary simplification and a specific definition of shock will always influence the prognostic value of a single shock parameter. As long as there is no standardized and broadly accepted definition of shock, validity of shock parameters will vary.

Additional to the above-mentioned problems, there are some more limitations that should be considered. The presented study is a retrospective analysis with its inherent limitations. Moreover, 71 of 843 patients (8.4%) had to be excluded due to missing data, which is in line with comparable studies [8, 18]. However, missing data can be misleading and might influence risk prediction [19].

All patients in this analysis were treated in a level I trauma center in a high-income country with a sophisticated trauma system. Since outcome parameters were precisely defined and all analyses were based on validated methods, our results may be generalized to countries with comparable medical standards.

### Conclusion

In conclusion, admission blood glucose is an easy to use point-of-care test that when combined with other parameters might help to identify multiply injured patients at particular risk of shock and mortality.

## References

[1] Sauaia A, Moore FA, Moore EE, Moser KS, Brennan R, Read RA, Pons PT (1995) Epidemiology of trauma deaths: a reassessment. J Trauma 38(2), 185–193. 7869433 10.1097/00005373-199502000-00006

[2] Mutschler M, Nienaber U, Munzberg M, Wolfl C, Schoechl H, Paffrath T, Bouillon B, Maegele M, TraumaRegister DGU (2013) The Shock Index revisited – a fast guide to transfusion requirement? A retrospective analysis on 21,853 patients derived from the TraumaRegister DGU. Crit Care 17(4), R172. 23938104 10.1186/cc12851 PMC4057268

[3] Hartmann B, Weise H, Bassenge E (1988) [Quality assurance in Riva-Rocci blood pressure measurement: simultaneous sphygmomanometry with open and covered pressure display]. Z Kardiol 77(8), 537–542. 3176598

[4] Cabedo Garcia VR, Silvestre Ramos G, Garcia Raimundo R, Ripoll Perello J, Hernandez Aguado I (1995) [The validity and reliability of arterial pressure measurement in a health center]. Aten. Prim. 15(1), 15–20. 7880949

[5] Mutschler M, Nienaber U, Munzberg M, Fabian T, Paffrath T, Wolfl C, Bouillon B, Maegele M (2014) Assessment of hypovolaemic shock at scene: is the PHTLS classification of hypovolaemic shock really valid? Emerg Med J 31(1), 35–40. 23302502 10.1136/emermed-2012-202130

[6] Yendamuri S, Fulda GJ, Tinkoff GH (2003) Admission hyperglycemia as a prognostic indicator in trauma. J Trauma 55(1), 33–38. 12855878 10.1097/01.TA.0000074434.39928.72

[7] Kreutziger J, Schlaepfer J, Wenzel V, Constantinescu MA (2009) The role of admission blood glucose in outcome prediction of surviving patients with multiple injuries. J Trauma 67(4), 704–708. 19820574 10.1097/TA.0b013e3181b22e37

[8] Kreutziger J, Rafetseder A, Mathis S, Wenzel V, El Attal R, Schmid S (2015) Admission blood glucose predicted haemorrhagic shock in multiple trauma patients. Injury 46(1), 15–20. 25441172 10.1016/j.injury.2014.09.018

[9] Kreutziger J, Lederer W, Schmid S, Ulmer H, Wenzel V, Nijsten MW, Werner D, Schlechtriemen T (2018) Blood glucose concentrations in prehospital trauma patients with traumatic shock: a retrospective analysis. Eur J Anaesthesiol 35(1), 33–42. 29135535 10.1097/EJA.0000000000000733

[10] Arbeitsgemeinschaft der Wissenschaftlichen Medizinischen Fachgesellschaften (2016) S3-Leitlinie: Polytrauma / Schwerverletzten-Behandlung. 01.07.2016. Available from: https://www.awmf.org/uploads/tx_szleitlinien/012-019l_S3_Polytrauma_Schwerverletzten-Behandlung_2017-08.pdf; [cited 28.01.2018].

[11] Deutsche_Gesellschaft_für_Orthopädie_und_Unfallchirurgie (2012) Weißbuch Schwerverletztenversorgung: Empfehlungen zur Struktur, Organisation, Ausstattung sowie Förderung von Qualität und Sicherheit in der Schwerverletzten-Versorgung in der Bundesrepublik Deutschland. Stuttgart, Thieme.

[12] Gennarelli TA, Wodzin E, Medicine AftAoA (2008) Abbreviated Injury Scale 2005: Update 2008. Barrington, Association for the Advancement of Automotive Medicine.

[13] Baker SP, O’Neill B, Haddon W Jr, Long WB (1974) The injury severity score: a method for describing patients with multiple injuries and evaluating emergency care. J Trauma 14(3), 187–196. 4814394

[14] Corp I (2016) IBM SPSS Statistics for Windows. Armonk, NY, IBM Corp.

[15] Youden WJ (1950) Index for rating diagnostic tests. Cancer 3(1), 32–35. 15405679 10.1002/1097-0142(1950)3:1<32::aid-cncr2820030106>3.0.co;2-3

[16] Bundesärztekammer (BÄK), Kassenärztliche Bundesvereinigung (KBV), Arbeitsgemeinschaft der Wissenschaftlichen Medizinischen Fachgesellschaften (AWMF) (2013) Nationale VersorgungsLeitlinie Therapie des Typ-2-Diabetes – Langfassung, 1. Edition. Version 4. 2013 last updated: November 2014. Available from: www.dm-therapie.versorgungsleitlinien.de; [cited: 28.01.2018]; DOI: 10.6101/AZQ/000213.

[17] German Trauma Society (DGU) Committee on Emergency Medicine, Intensive Care and Trauma Management (Sektion NIS) of the German Trauma Society (DGU) (2017) Annual Report Trauma Register (TR-DGU) 2017. Available from: http://www.traumaregister-dgu.de/fileadmin/user_upload/traumaregister-dgu.de/docs/Downloads/TR-DGU_Annual_Report_2017.pdf [cited: 30.01.2018].

[18] Kreutziger J, Wenzel V, Kurz A, Constantinescu MA (2009) Admission blood glucose is an independent predictive factor for hospital mortality in polytraumatised patients. Intensive Care Med 35(7), 1234–1239. 19238355 10.1007/s00134-009-1446-z

[19] Trickey AW, Fox EE, del Junco DJ, Ning J, Holcomb JB, Brasel KJ, Cohen MJ, Schreiber MA, Bulger EM, Phelan HA, Alarcon LH, Myers JG, Muskat P, Cotton BA, Wade CE, Rahbar MH, Group PS (2013) The impact of missing trauma data on predicting massive transfusion. J Trauma Acute Care Surg 75(1 Suppl 1), S68–74. 23778514 10.1097/TA.0b013e3182914530 PMC3736742

